# Consanguineous marriages, premarital screening, and genetic testing: a survey among Saudi university students

**DOI:** 10.3389/fpubh.2024.1328300

**Published:** 2024-03-21

**Authors:** Tahir Jameel, Mukhtiar Baig, Manal Abdulaziz Murad, Zohair Jamil Gazzaz, Youssof Mal, Wedyan Eid Alyoubi, Ghadi Hamed Alyoubi, Shoug Tawfiq Alaslani, Hanan Abdullah Alshuaibi, Ayesha Nawaz, Turki Alkaabi

**Affiliations:** ^1^Department of Internal Medicine, Faculty of Medicine Rabigh, King Abdulaziz University, Jeddah, Saudi Arabia; ^2^Department of Clinical Biochemistry, Faculty of Medicine Rabigh, King Abdulaziz University, Jeddah, Saudi Arabia; ^3^Department of Family and Community Medicine, Faculty of Medicine in Rabigh, King Abdulaziz University, Jeddah, Saudi Arabia; ^4^Medical Students, Faculty of Medicine Rabigh, King Abdulaziz University, Jeddah, Saudi Arabia; ^5^SHO, Gold Coast University Hospital, Southport, QLD, Australia

**Keywords:** consanguinity, genetic disorders, thalassemia, sickle cell anemia, social factors, premarital counseling, genetic testing

## Abstract

**Background:**

Marriage among cousins or close relatives, i.e., consanguinity, is prevalent in many parts of the world, especially the Muslim world. Across civilizations, cultural norms, religious beliefs, and economic factors affect consanguineous marriages (CMs); however, such marriages have social, genetic, and health repercussions. The present study investigated the university students’ attitudes regarding CMs and factors influencing their attitudes at King Abdulaziz University (KAU), Jeddah, Kingdom of Saudi Arabia (KSA).

**Methods:**

This cross-sectional prospective study was conducted at KAU Jeddah in 2023. The questionnaire was distributed via electronic media (Emails, Facebook Messenger & WhatsApp). The convenience sampling technique was used to select participants, and descriptive and inferential statistics were used to analyze the data on SPSS-26.

**Results:**

A total of 1707 university students were part of the study (females, 1,198, 70.2%; males, 509, 29.8%). Almost half of the participants, 819 (48.0%), had parents with CMs. Most of the participants, 1,391 (81.5%), had CMs in the family. Half of the participants disagreed that parents consider marriage stable due to high compatibility and the same social relationship before and after marriage. About one-third of respondents said parents believe family marriage transmits cultural values and continuity and keeps wealth in the family. More than three-fourths of the participants stated that if marriage is arranged with first cousins, they will opt for genetic analysis (82.5%) and premarital counseling (85.2%). The personal attitudes of females (*p* < 0.001), undergraduate (*p* = 0.02), and health sciences students (*p* = 0.02) were more positive than their counterparts. Males (OR = 0.41; *p* < 0.001) and non-health sciences students (OR = 0.68; *p* = 0.01) were less likely to have significant positive attitudes than their counterparts. Among participants who had CM parents, males (OR = 0.397; *p* < 0.001) and non-health sciences students (OR = 0.60; *p* = 0.01) and urban residents (OR = 0.59; *p* = 0.01) had significantly lower odds of having a positive attitude than their counterparts.

**Conclusion:**

The practice of CMs is still prevalent in Saudi culture, with almost half of the participants having CM parents and the majority reporting these marriages in their families. Personal attitudes toward CMs were extremely positive. Most students prefer genetic testing and premarital counseling if marrying first cousins. Gender, faculty, parental income, and educational background influenced participants’ attitudes.

## Introduction

Consanguinity describes a marriage arrangement between males and females, related to each other through ancestry (1). Such marriages have occurred in many societies and have social, genetic, and health implications. Because their children would be inheriting two copies of harmful recessive alleles from closely related parents, genetic abnormalities and inherited disorders are expected to be frequently seen in them (2). These marriages might perpetuate family social and economic disparities since they may be driven by tradition or economics rather than personal choice. Consanguineous marriages (CMs) acceptance and prevalence are influenced by cultural norms, religious beliefs, and economic considerations across civilizations. There is a debate on balancing cultural norms with such marriage’s health and socioeconomic consequences (3). Consanguinity is common in Africa, the Middle East, the Indian subcontinent, Pakistan, Bangladesh, and Iran, especially in Muslim-majority areas (4).

CM is preferred in many communities because marrying a close relative helps improve family relationships and preserve cultural values. The family elders can be consulted in a major disagreement, and the problem can be resolved. However, there are many risks involved. Consanguinity can increase the chance of genetic diseases in offspring, especially if the population is ethnically similar (5). Blood disorders like sickle cell anemia and thalassemia are common in countries like the Kingdom of Saudi Arabia (KSA), the population of the Mediterranean basin, India, Pakistan, Bangladesh, Southeast Asia & Pacific Islands (6). Mental illnesses like depression and schizophrenia may also be more prevalent in families that practice CM (7). Despite efforts to educate people about the risks, many continue to marry within their families. According to a recent survey, two-thirds of educated participants practicing CM were aware that there are transmissible genetic variables associated with this practice. However, only a few were aware of diagnostic facilities available for post-conceptual testing (8).

Medical professionals, teachers, intellectuals, and the media are the main sources of knowledge and awareness regarding CMs (9). Premarital screening of the expected couple can also indicate whether an illness or disease may be inherited, and individuals can be informed of their conditions and the effects of their diseases. Despite the general rise in awareness and the introduction of premarital testing in 2003 in KSA, reports continued to point out the significant incidence of CMs in KSA (10). A study pointed out that despite high levels of education, participants had little understanding of the relationship between consanguinity and genetic illnesses (9).

Premarital screening is an amazingly effective tool to prevent various genetic disorders in societies with a high incidence of consanguinity. It is a form of advice offered to the expected couple regarding the chance of transmission of profoundly serious and debilitating disorders like thalassemia, sickle cell disease, and other hemoglobinopathies in the coming generations (10). It offers the couple and both families an option to be fair with each other and a way to prevent future conflicts and unexpected hardships (11). Premarital counseling and genetic testing in a consanguineous couple aims to predict and diagnose unrecognized diseases and reduce the transmission of disorders that may damage future generations’ health (12).

Assessing university students’ knowledge, concepts, and attitudes is crucial in a society with a high prevalence of consanguinity. They are the decision-makers and parents of the future (13). The importance of the study rests in addressing a variety of issues, ranging from the awareness regarding CM to the accompanying health hazards, involving the role of education and cultural influences in shaping public opinion. The current study findings could help shape healthcare policies and genetic counseling services, thus improving public health and the availability of genetic testing to the affected couples. The primary objective of the current study is to assess university students’ attitudes regarding CMs and factors influencing their attitudes at King Abdulaziz University (KAU), Jeddah, KSA.

## Methods

The prospective cross-sectional study was carried out from March 15 to June 15, 2023, at KAU, Jeddah, KSA. The project was approved by the Ethical Review Committee of the university (Reference No 316-23). Only KAU students were invited to participate in this study. At the beginning of the online questionnaire, all the participants were informed about the research objectives, and a statement about participants’ consent was given; it was stated that their completion of the online questionnaire would be deemed their consent to participate in the study. Additionally, our data collection was strongly in line with the protocol related to institutional and national ethical standards, as well as the Helsinki Declaration. The date was kept strictly anonymous, and confidentiality was maintained. The representative sample size was calculated using the Raosoft sample size calculator. The calculated sample size was 382, taking the population proportion of positive attitudes to 50%. The margin of error was kept at 5% and a confidence level of 95%. However, we inflated the sample size to generalize the results.

Our primary outcome variable was the participants’ personal attitudes (PAs), and our secondary outcome variable focused on the PAs of participants whose parents had entered CMs. The hypothesis posited that the PAs of study participants could be influenced by various factors, such as age, gender, educational status, type of family, the source of information about consanguinity, parents’ educational background, income, and CM, and the source of information about consanguinity. To test this hypothesis, we employed the Chi-square test and binary logistic regression analysis, evaluating the relationship of these variables with both primary and secondary outcomes.

### Data collection procedure

Two thousand male and female students from KAU were invited from all the approachable faculties, and participants were selected using the convenience sample technique. The questionnaire was distributed via electronic media (Emails, Facebook Messenger & WhatsApp). Of 2000 students, 1707 responded to our questionnaire, bringing the response rate to 85.35%. As the questionnaire link was sent via multiple platforms, there was a risk of double entry by participants; thus, the following precautions were implemented to limit this risk. The same questionnaire link was sent across various platforms; however, at the start of the online questionnaire, a brief paragraph outlining the research was provided, and the primary investigator’s name, email ID, and contact number in case of any questions. So, at the start, all participants knew whether they had completed the questionnaire. Furthermore, respondents were clearly instructed to complete the questionnaire just once, regardless of the platform utilized, and the researchers set up limits within the survey platform to limit the number of times a respondent may submit the form. The researchers also regularly cleaned data to identify and remove duplicate entries.

### Data collection instrument

The questionnaire was constructed through Google Forms to collect the participants’ responses regarding consanguinity. Several questions were taken from a previously published study (14). The questionnaire was prepared in both English & Arabic language for a better understanding of the questions by the study participants. It was ensured that our questionnaire evaluated the concepts and attitudes of our participants along with the freedom to express their personal opinions on the delicate topic. Two senior faculty members and a medical educationist evaluated the questionnaire’s content and construct validity. A language expert translated the English questionnaire to Arabic and back to English to ensure the comprehension of the Arabic translation and to remove any ambiguity.

As a pilot project, fifty students were asked to fill in the questionnaire, and changes were made to the contents to ensure a better understanding of all the required points by the participants. Moreover, the reliability of the questionnaire was checked by Cronbach’s alpha, and it was found to be 0.69 for the community attitudes.

The questionnaire consisted of the demographic section with questions regarding age, gender, marital status, history of consanguinity in the family, social status, education level of both parents and their occupation, level of study, etc. In the community attitude section, questions were asked regarding several factors favoring consanguinity, like which points are mostly considered by parents when deciding regarding the marriage of their children. In the personal attitudes section, students were asked about their choices if they were given the option to decide on their marriage. Would they prefer to marry a first cousin or an unrelated family? If a CM is decided, would our study respondents prefer premarital counseling and genetic analysis of both partners’ or would they be going against it as per family traditions?

Questions also included regarding the knowledge of diseases associated with consanguinity.

There were several types of questions, such as “yes and no,” “yes, no and unsure,” and 3-point Likert scale questions were used to assess community attitudes.

Attitude scoring system: PA was scored as yes = +1 score, no = −1 score. The plus score was regarded as positive, while the 0 or minus score was regarded as negative (15). To answer the question, “If you were in a position to decide on your marriage, would you opt for marriage with a first cousin?” The answers “No” and “Yes” were scored in reverse order. In the case of community attitude (CA), 0 was allocated to disagree, 1 to neutral, and 2 to agree. The positive attitude toward CMs indicates that those students’ responses did not favor CM and preferred genetic testing and premarital screening in the case of CM. In contrast, the negative attitude mirrored the opposite.

### Statistical analysis

SPSS-26 was utilized for the evaluation of the collected data. Different variables were analyzed for frequencies and percentages for the responses. Chi-square and two-proportion testing were conducted to compare personal attitudes (positive and negative) with other variables. Binary logistics regression was used to explore the association of variables with PA. Significant *p*-values represented <0.05.

## Results

There were 1707 participants in the study (females 1,198, 70.2%; males 509, 29.8%), and the mean age of the participants was 22.3 years. Almost half of the participants, 819 (48%), had parents with CMs. Most of the participants, 1,391 (81.5%), had CMs in the family. Table 1 shows all the details of the study participants.

**Table 1:** Demographic characteristics of the study participants.

Age (Years)	Frequency (*n* = 1707)	Percent
18–21	1,102	64.6
26–29	54	3.2
≥ 30	87	5.1
Gender		
Male	509	29.8
Female	1,198	70.2
Nationality		
Saudi	1,550	90.8
Non-Saudi	157	9.2
Enrolled in		
Graduate study	383	22.4
Undergraduate study	1,324	77.6
Faculty		
Health sciences	804	47.10
Non-health sciences	903	52.90
Area of residence		
Rural	259	15.2
Urban	1,448	84.8
Family members’ occupations related to healthcare		
No	996	58.3
Yes	711	41.7
Marital status		
Divorced	9	0.5
Married	151	8.8
Unmarried	1,547	90.6
Parents monthly income ^*^ (Saudi Riyal)		
< 5,000	354	20.7
5,000–10,000	401	23.5
10,001–15,000	313	18.3
15,001–20,000	294	17.2
>20,000	345	20.2
Father’s education level		
No formal education	45	2.6
Primary	115	6.7
Secondary	174	10.2
Graduate	429	25.1
Postgraduate	944	55.3
Mother’s education level		
No formal education	99	5.8
Primary	145	8.5
Secondary	158	9.3
Graduate	391	22.9
Postgraduate	914	53.5
Father occupation		
Government	663	38.8
Private Job	277	16.2
Retired	557	32.6
Farmer	20	1.2
Business	190	11.1
Type of family		
Nuclear Family	955	55.9
Joint Family	752	44.1
Source of information about consanguinity		
Family member	859	50.3
Social Media	573	33.6
Internet Source	269	15.8
University/College	6	0.4
Parents with consanguineous marriage		
No	888	52.0
Yes	819	48.0
If your parents have a consanguineous marriage, which category of cousins do they belong to?		
Distant cousins	58	3.4
First cousins	342	20.0
Second cousins	419	24.5
Consanguineous marriage in the family		
No	316	18.5
Yes	1,391	81.5
Which of the following diseases can be detected by premarital screening?		
Sexually transmitted	1,092	64.0
Genetic Disorders	510	29.9
Inherited metabolic disorder	55	3.2
Infective viral disorder	50	2.9

^*^One Saudi Riyal is equal to 0.27 USD.

The analysis of community attitude (CA) of the study participants revealed that only one-fifth of the participants agreed to the consideration of parents regarding the high chance of a stable marriage because of better compatibility & continuity of the same social relationship in the pre-and post-marriage period while deciding regarding their children’s marriage in the family and half of the participants disagreed. Almost one-third of the participants agreed that parents consider that family marriage helps transmit cultural values and cultural continuity, and wealth or property will remain within the family. More than 40% of the participants disagreed (Table 2).

**Table 2:** Frequency of responses regarding community and personal attitude (*n* = 1707).

Statements	Frequency	Percent
Community attitude (CA)		
Do you agree that while deciding regarding their children’s marriage in the family, parents consider that there is a high chance of stability in marriage due to high compatibility?		
Disagree	846	49.6
Neutral	488	28.6
Agree	373	21.9
Do you agree that while deciding regarding their children’s marriage in the family, parents consider that there will be the same social relationship before and after the marriage?		
Disagree	879	51.5
Neutral	458	26.8
Agree	370	21.7
Do you agree that while deciding regarding their children’s marriage in the family, parents consider that it helps in the transmission of cultural values and cultural continuity?		
Disagree	766	44.9
Neutral	401	23.5
Agree	540	31.6
Do you agree that the girl’s parents prefer to have their daughter living near them and to enjoy the presence of their grandchildren?		
Disagree	267	15.6
Neutral	361	21.1
Agree	1,079	63.2
Do you agree that while deciding their children’s marriage in the family, parents consider that wealth or property will remain within the family?		
Disagree	687	40.2
Neutral	497	29.1
Agree	523	30.6
Do you agree that while deciding their children’s marriage in the family, parents consider that they can influence both partners in case of any disagreement?		
Disagree	455	26.7
Neutral	342	20.0
Agree	910	53.3
Personal attitude (PA)		
If you were in a position to decide on your marriage, would you opt for marriage with a first cousin?		
No	1,454	85.2
Yes	253	14.8
If your marriage is arranged with one of your first cousins, would you opt for premarital counseling?		
No	298	17.5
Yes	1,409	82.5
If your marriage is arranged with one of your first cousins, would you opt for the genetic analysis of both partners?		
No	119	7.0
Yes	1,588	93.0
In your opinion, law and regulation should ban consanguineous marriage if there is a serious chance of having a child affected with a genetic disease?		
No	311	18.2
Yes	1,396	81.8

For scoring the PA of our study participants, the analysis of PA showed that more than 80% of the participants would not opt for marriage with a first cousin. More than three-fourths of the participants stated that if marriage is arranged with first cousins, they will opt for genetic analysis (82.5%) and premarital counseling (85.2%) (Table 2).

A comparison of PA (positive and negative) with demographics of categorical factors revealed that 18–21 years and 26–29 years had more positive attitudes than other age groups (*p* = 0.03), and married and unmarried had more positive attitudes than divorced (*p* = 0.03). Females outperformed males in terms of positive scores (*p* < 0.001). Undergraduate (*p* = 0.02) and health sciences (*p* = 0.002) students were more positive than their counterparts. Students with postgraduate fathers had more positive attitudes (*p* = 0.03), but students with moms who had no formal education had more negative attitudes (*p* = 0.02) compared to other educational groups (Table 3).

**Table 3:** Comparison of personal attitude (positive and negative) with demographics of categorical variables (*n* = 1707).

Variables	Responses	Personal attitude score		*p*-value^**^
		Negative attitude *N* (%)	Positive attitude *N* (%)	
Age (years)	18–21	126 (11.4)	976 (88.6)	0.03^*^
	22–25	70 (15.1)	394 (84.9)	
	26–29	12 (22.2)	42 (77.8)	
	≥ 30	14 (16.1)	73 (83.9)	
Marital status	Divorced	2 (22.2)	7 (77.8)	0.04^*^
	Married	29 (19.2)	122 (80.8)	
	Unmarried	191 (12.3)	1,356 (87.7)	
Gender	Male	122 (24)	387 (76)	<0.001
	Female	100 (8.3)	1,098 (91.7)	
Nationality	Saudi	194 (12.5)	1,356 (87.5)	0.06
	Non-Saudi	28 (17.8)	129 (82.2)	
Enrolled in	Graduate	63 (16.4)	320 (83.6)	0.02^*^
	Undergraduate	159 (12)	1,165 (88)	
Area of residence	Rural	35 (13.5)	224 (86.5)	0.76
	Urban	187 (12.9)	1,261 (87.1)	
Type of family	Nuclear family	114 (11.9%)	841 (88.1)	0.08
	Joint family	108 (14.4)	644 (85.6)	
Faculty	Health sciences	83 (10.3)	721 (89.7)	0.002^*^
	Non-health sciences	139 (15.4)	764 (84.6)	
Monthly income (Saudi Riyal)^***^	<5,000	61 (17.2)	293 (82.8)	0.09
	5,000–10,000	46 (11.5)	355 (88.5)	
	10,001–15,000	40 (12.8)	273 (87.2)	
	15,001–20,000	38 (12.9)	256 (87.1)	
	>20,000	37 (10.7)	308 (89.3)	
Education of father	No formal education	10 (22.2)	35 (77.8)	0.03^*^
	Primary	21 (18.3)	94 (81.7)	
	Secondary	27 (15.5)	147 (84.5)	
	Graduate	59 (13.8)	370 (86.2)	
	Postgraduate	105 (11.1)	839 (88.9)	
Education of mother	No formal education	22 (22.2)	77 (77.8)	0.02^*^
	Primary	22 (15.2)	123 (84.8)	
	Secondary	25 (15.8)	133 (84.2)	
	Graduate	44 (11.3)	347 (88.7)	
	Postgraduate	109 (11.9)	805 (88.1)	
Job of father	Government	80 (12.1)	583 (87.9)	0.36
	Private Job	30 (10.8)	247 (89.2)	
	Retired	83 (14.9)	474 (85.1)	
	Farmer	4 (20)	16 (80)	
	Business	25 (13.2)	165 (86.8)	
Job of mother	Government	68 (13.6)	433 (86.4)	0.50
	Private job	19 (16.4)	97 (83.6)	
	Retired	13 (10.5)	111 (89.5)	
	Housewife	113 (12.3)	802 (87.7)	
	Business	9 (17.6)	42 (82.4)	
Source of information about consanguinity	Family member	112 (13)	747 (87)	0.17
	Social media	81 (14.1)	492 (85.9)	
	Internet source	27 (10)	242 (90)	
	University/College	2 (33.3)	4 (66.7)	
Parents have consanguineous marriage	No	105 (11.8)	783 (88.2)	0.15
	Yes	117 (14.3)	702 (85.7)	
Consanguineous marriage in family	No	40 (12.7)	276 (87.3)	0.46
	Yes	182 (13.1)	1,209 (86.9)	

^*^p-value ≤0.05 was considered significant. ^**^Chi-square test was applied. ^***^One Saudi Riyal is equal to 0.27 USD.

Males (OR = 0.41; *p* < 0.001) and non-health sciences students (OR = 0.68; *p* = 0.01) were less likely to have significant positive attitudes than their counterparts. Individuals with parents’ monthly incomes ranging from 5,000 to 10,000 Saudi Riyals had higher odds of having a positive PA than those with less than 5,000 Saudi Riyals (OR = 1.60; *p* = 0.03) (Table 4).

**Table 4:** Association of variables with personal attitudes (binary logistics regression).

Parameters vs. PA Score achieved^*^	Responses	Beta coefficient	Odds Ratio	*p*-value	95% C.I. for Odds Ratio	
					Lower	Higher
Age	18–21	-	Reference			
	22–25	−0.105	0.900	0.574	0.624	1.299
	26–29	−0.360	0.698	0.374	0.315	1.544
	≥ 30	0.246	1.279	0.557	0.563	2.905
Gender	Female		Reference			
	Male	−0.891	0.410	<0.001^*^	0.264	0.636
Nationality	Saudi		Reference			
	Non-Saudi	−0.076	0.927	0.759	0.572	1.503
Enrolled in	Graduate		Reference			
	Undergraduate	0.106	1.112	0.630	0.723	1.709
Studying in faculty	Health Sciences		Reference			
	Non-Health Sciences	−0.378	0.686	0.015^*^	0.506	0.929
Residence	Rural		Reference			
	Urban	0.131	1.140	0.528	0.758	1.716
Marital status	Divorced		Reference			
	Married	−0.077	0.926	0.930	0.166	5.149
	Un-married	0.630	1.877	0.464	0.348	10.117
Parents monthly income (Saudi Riyal)^***^	< 5,000		Reference			
	5,000–10,000	0.476	1.609	0.030^*^	1.048	2.472
	10,001–15,000	0.362	1.436	0.120	0.910	2.267
	15,001–20,000	0.260	1.297	0.270	0.817	2.058
	>20,000	0.363	1.437	0.124	0.906	2.282
Type of family	Nuclear		Reference			
	Joint	−0.061	0.941	0.688	0.700	1.266
Parents have consanguineous marriage	No		Reference			
	Yes	−0.240	0.787	0.117	0.583	1.062
Consanguineous marriage in the family	No		Reference			
	Yes	−0.034	0.966	0.863	0.654	1.427

^*^Dependent variable is Personal attitude (PA) (Positive and Negative). ^**^p-value ≤0.05 was considered significant. ^***^One Saudi Riyal is equal to 0.27 USD.

Among participants with parents having CMs, Males (OR = 0.397; *p* < 0.001) and non-health sciences students (OR = 0.60; *p* = 0.01) and urban residents (OR = 0.59; *p* = 0.01) have significantly lower odds of having positive attitudes than their counterparts. Conversely, individuals with parents’ monthly income ranging from 5,000 to 10,000 Saudi Riyals had higher odds of having positive personal attitudes than those with less than 5,000 Saudi Riyals (OR = 1.84; *p* = 0.04) (Table 5).

**Table 5:** Association of variables with personal attitudes of participants who have parents with consanguineous marriage (binary logistics regression analysis).

Parameters vs. personal attitude of participants who have parents with consanguineous marriage^*^		Beta coefficient	Odds Ratio	*p*-value^**^	95% C.I. for Odds Ratio	
					Lower	Upper
Age	18–21		Reference			
	22–25	0.067	1.070	0.64	1.788	0.640
	26–29	0.365	1.440	0.41	5.036	0.412
	≥ 30	0.811	2.251	0.67	7.480	0.677
Gender	Female		Reference			
	Male	−0.923	0.397	<0.001^*^	0.259	0.611
Nationality	Saudi		Reference			
	Non-Saudi	−0.369	0.692	0.30	0.343	1.396
Enrolled in	Graduate		Reference			
	Undergraduate	0.035	1.035	0.91	0.567	1.891
Faculty	Health sciences		Reference			
	Non-health sciences	−0.519	0.60	0.01^*^	0.394	0.913
Residence	Rural		Reference			
	Urban	−0.106	0.899	0.01^*^	0.512	1.580
Marital status	Divorced		Reference			
	Married	0.267	1.306	0.78	0.198	8.631
	Un-married	1.18	3.281	0.20	0.534	20.171
Parents monthly income (Saudi Riyal)^***^	< 5,000		Reference			
	5,000–10,000	0.610	1.840	0.04^*^	1.017	3.329
	10,001–15,000	0.376	1.457	0.22	0.789	2.689
	15,001–20,000	0.363	1.437	0.27	0.750	2.755
	>20,000	0.362	1.437	0.26	0.762	2.711
Type of family	Nuclear		Reference			
	Joint	−0.211	0.810	0.31	0.537	1.220

^*^Dependent variable is Personal attitude (PA) (Positive and Negative). ^**^p-value ≤0.05 was considered significant. ***One Saudi Riyal is equal to 0.27 USD.

## Discussion

Consanguinity or marriage among close blood relatives might dramatically increase the chance of many health problems, ranging from various cognitive difficulties, heart defects, impaired hearing, and several autosomal disorders in future generations (16). Understanding the negative repercussions of such partnerships and the perspectives of younger people plays a critical role in continuing or discouraging this practice (17). The present study found almost half of the participants (48%) had parents with a history of CMs. A few studies have shown varying degrees of prevalence of consanguinity in KSA (18–20). El Hazmi et al. reported a CM prevalence of 57.7% (18). Another study showed the prevalence of consanguinity in 56% of all marriages in Saudi Arabia (19). However, results showed that the incidence was higher in rural areas than in urban populations (18, 19). A study conducted in Riyadh, Saudi Arabia, on a relatively small group of educated couples stated that 39.8% of CMs in their selected group (20). A recent cross-sectional study mentioned a 40% prevalence of CMs in Albaha province in KSA (21). They mentioned an astounding result: CM prevalence was higher among their study participants than their parents (40% vs. 31%). This is an alarming situation. It indicates that with time, the incidence of CMs is increasing in certain areas. Ultimately, this increasing trend will increase the adverse consequences of CMs in society. So, more healthcare budgets and resources will be diverted to manage the diseased children of the parents of CMs.

There is a need for nationwide awareness efforts on this important and growing problem. Once the younger society members acknowledge the hazards of a traditional happening, they will be in a better position to spread this awareness among close family members and friends (22).

The current study results showed that most of our study participants had a clear idea of the association of consanguinity with various inherited disorders. This attitude should be reflected in society’s cumulative thinking. However, changes in a society’s attitude take longer, but the positive effects can be observed in one or two generations (23). Regarding our study subjects’ knowledge regarding the scope of premarital screening, about one-third of the participants were sure that certain genetic disorders could be detected in future couples. Al Ahdal et al. published a similar study regarding university students of Riyadh city. They mentioned high levels of awareness regarding consanguinity and genetic disorders (24).

The current study observed from the demographic data that most participants’ parents were highly educated even though the CMs were prevalent among the family. A similar observation was mentioned in a recent study when they targeted only educated couples and found that almost 40% of them were married to close relatives, especially their first cousins. The age of individuals and education did not affect consanguinity (20). A study explored various determinants of consanguinity among the Arab population and concluded that there is a complex relationship between consanguinity and socioeconomic culture in different tribes and localities. Education was not an essential determinant factor in marriage-related decisions (25).

Almost half (49.6%) of the participants disagreed with the common consideration regarding parents’ perspectives on CMs. They disagreed that parents favor the CMs because of better compatibility and continuation of the same social culture. A Turkish study reported that the community attitude develops with maturity (26). Studies from Turkey & Iran had the impression that in the premarital stage, the opinions are not mature enough compared to the mature age when one faces the hardships of life (26, 27). Our findings regarding community attitude are similar to those of a Qatari study that reported a similar attitude to this aspect of the determinant (28). In contrast, while taking the opinion of parents agreeing with the CM of their children, a Pakistani study pointed out that cultural continuity and wealth safety are considered important factors when deciding their children’s marriages (29).

Similar to the present results, a study in UAE targeting CM women confirmed that parents may accept their son living away, but keeping their daughter in close vicinity is a strong wish of the parents (30).

The present study revealed that females, undergraduate, and health sciences students exhibited more positive attitudes (against consanguineous marriages) than their counterparts. Students with postgraduate fathers had positive attitudes against CMs, but students with mothers who had no formal education had negative attitudes (inclined toward CM) compared to other educational categories. Education, awareness, and a better understanding of genetic risks, especially among health sciences students, may contribute to students’ positive attitudes toward CMs. Cultural developments and shifting societal standards may also play a role in these attitudes. Furthermore, students with postgraduate fathers had more positive attitudes due to their better educational backgrounds. Education can help promote an informed and open-minded perspective, as well as raise awareness of the challenges related to consanguinity. Conversely, individuals whose mothers were uneducated may be more influenced by conventional ideas. Socioeconomic factors may also affect these disparities, as families with postgraduate dads tend to have higher socioeconomic status, influencing their opinions. On the other hand, negative attitudes among students whose mothers have no education may be associated with a lower socioeconomic standing. A Saudi study’s findings indicated that the prevalence of CM was most pronounced among the daughters of CM parents, reaching 52.27%. It showed that CM’s parents preferred CM for their daughters (24). Some researchers mentioned that consanguinity had been common in Arab nations ever before the dawn of Islam. In contrast, in most European and Eastern inhabitants, first cousins are considered real siblings (31, 32).

A study discussed the state measures adopted by various countries and law enforcement agencies like the US, China, and Greece, where first-cousin marriage was legally prohibited with the verdict from the Church (33). It led to drastic changes in the incidence of CMs, resulting in a decrease in thalassemia/ sickle cell disease in those areas. Other researchers have also mentioned this wonderful achievement (34).

In our study, most responses negated various concepts of parents’ influence on the children’s marriage. A recent Omani study observed that parents, family, and culture are strong factors in continuing the consanguinity in young Arab generations (35). Children’s marriages are traditionally decided in certain tribes as early as the birth of a baby. Females are often married to very young children to keep the family’s wealth and properties within the family. CMs are traditionally considered safe marriages where the elders can intervene at difficult times (36).

A vast majority of our participants were unmarried. A very recent study concluded that young unmarried individuals are influenced toward consanguinity due to many determinants, such as family traditions, parents’ influence, and non-serious attitudes because of age (35). But now, the times are changing very rapidly; while responding to the questions about PA, our participants responded very maturely, and 81.8% of the participants believed that if there is a risk of any dire consequences in the coming generations due to consanguinity, this practice should be banned by law. Similar changing concepts of the younger generation toward the CM ban were reported by an Indian and Saudi study (36, 37).

The attitudes of the present study participants were commendable, with over 80% expressing remarkably positive views. Specifically, more than 80% of respondents opposed first-cousin marriage and advocated for premarital counseling and genetic screening in situations involving consanguinity, even when facing various pressures. Most respondents supported a legal ban on CMs. It is a known fact that in children born due to CM, and especially among first cousins, there is an additional risk of 1.7–2.8% for multiple congenital disabilities and autosomal recessive disorders (38, 39). In couples with no known genetic disorders in the family, there is an additional risk in the offspring of first cousins compared to the general population. A British study mentioned positive PA among younger students who were well aware of health hazards in their offspring due to CMs (40). An Indonesian study mentioned similar expressions of personal attitudes among Indonesian youth (41). Thain et al. mentioned the willingness of 13 consanguineous couples who opted for genetic screening early after conception (42). A study from Sudan reported that 82.3% of couples in Sudan volunteered for genetic screening after their CM (43). In many of them, therapeutic abortions were recommended because of serious problems in the conceptus.

Our study results suggested that positive attitudes against CMs are associated with certain factors such as female gender, health sciences students, and parents’ monthly incomes among all participants and remain consistent among those with parents who have CMs. There could be several possible explanations for these associations. Females may feel more empowered and autonomous, especially in environments where gender norms are changing. This empowerment may result in positive attitudes against old practices such as CMs. Health sciences students may have better access to knowledge concerning the potential genetic hazards connected with consanguinity. This improved knowledge may help shape their attitudes. Parents’ higher monthly salaries may be related to better financial security and autonomy. Individuals from higher-income households may be more likely to make independent decisions, including expressing negative opinions toward CMs. People with consanguineous parents oppose CM because they know the difficulties or complexities that may arise. More research, including qualitative investigations, could provide light on the underlying causes of these connections. As CM is common in the Kingdom, it was expected that people would have good knowledge and attitudes about it. However, a good attitude should be reflected in the society practice. Still, one hopes that continuing awareness efforts are the only way to improve community attitudes and that, with time, the incidence of CMs will decrease substantially. A couple of studies categorically mentioned this mood and trend of the younger generation to discourage consanguinity in society so that the coming generation is free from the number of prevalent autosomal recessive genetic disorders (42–44). An Indian study has suggested increasing awareness of congenital problems in society due to continuing CMs (45).

Several recommendations emerge from our research findings. This includes educating people on the dangers of CMs, benefits of premarital screening, and genetic testing. Secondly, the institutions should plan to provide information on genetic counseling & other essential information to students, especially those who are in or planning CMs. Thirdly, use of mass media and social media to initiate campaigns and continue them regularly to make them aware of the harmful effects of cousin marriages, the importance of premarital screening, and genetic testing.

### Implications of findings to the practice of public health and disease prevention

Despite our study findings showing good positive attitudes among university students, adding health education on consanguinity and genetic risks into educational curricula can be a long-term strategy. This integration guarantees that future generations are educated and competent to make sensible marriage and family planning decisions. The observed predictors of positive attitudes, such as gender, faculty, parents’ income, and educational background, highlight the significance of adapting interventions to specific population groups. Customized public health initiatives can target the specific needs, concerns, and perceptions of various populations, increasing the effectiveness of preventative actions. As the consanguinity is deeply rooted in Saudi culture, interventions must be culturally sensitive and respectful. In this regard, collaboration with religious and community leaders and educational institutions can help disseminate health information while maintaining cultural values.

Our findings suggest the importance of developing or strengthening policies that address the possible health consequences of CMs. Public health experts can lobby for and work with lawmakers to implement or improve legislation regarding CMs. To address the complicated issue of CMs and decrease health risks in KSA, various public health methods that integrate education, counseling, policy development, and cultural sensitivity are needed.

Long-term studies are required to assess the efficacy of initiatives to tackle the CMs in society. In this regard, international collaboration might assist in sharing best practices.

### Limitations

This study has several limitations, like all other cross-sectional investigations. The survey mainly included university students, who may not represent the Saudi population. Saudi Arabia is culturally and geographically diverse. The study may have disregarded regional variances in attitudes about consanguineous marriage, which might vary considerably. The convenience sample of university students in the study may introduce sampling bias. This sample may not represent the Saudi population, especially non-university students. Because of the culturally sensitive topic of CM, respondents may have concealed their true feelings, resulting in social desirability bias and underreporting. The study relies on quantitative data and does not provide qualitative insight into participants’ CM attitudes and perceptions.

## Conclusion

The CM practice remains firmly established in Saudi culture, with almost half of the participants having parents with CMs, and the majority reported these marriages in their families. Concerning CMs, personal attitudes were highly positive. Most of the students responded they would choose genetic analysis and premarital counseling if they were to marry first cousins. Gender, faculty, parents’ income, and educational background emerged as significant factors influencing participants’ attitudes.

These results emphasize the need for targeted educational and awareness programs to promote informed decision-making about CMs and address cultural norms and health repercussions. More research and policy considerations are needed to solve this complicated societal challenge that continues to evolve.

## Data availability statement

The original contributions presented in the study are included in the article/Supplementary material, further inquiries can be directed to the corresponding author.

## Ethics statement

The present study was approved by the Ethical Review Committee of the King Abdulaziz University, Jeddah, Saudi Arabia (Reference No. 316-23). The study was conducted in accordance with local legislation and institutional requirements. The ethics committee/institutional review board waived the requirement of written informed consent for participation or the participants’ legal guardians/next of kin because at the beginning of the online questionnaire, all the participants were informed about the research objectives and a statement about participants’ consent was given; it was stated that their completion of the online questionnaire would be deemed their consent to participate in the study.

## Author contributions

TJ: Conceptualization, Supervision, Writing – original draft, Writing – review & editing. MB: Conceptualization, Formal analysis, Investigation, Writing – review & editing. MM: Formal analysis, Methodology, Project administration, Writing – review & editing. ZG: Investigation, Project administration, Supervision, Writing – review & editing. YM: Formal analysis, Methodology, Supervision, Writing – review & editing. WA: Conceptualization, Investigation, Methodology, Writing – review & editing. GA: Conceptualization, Investigation, Methodology, Writing – review & editing. SA: Conceptualization, Investigation, Project administration, Writing – review & editing. HA: Conceptualization, Investigation, Project administration, Writing – review & editing. AN: Conceptualization, Methodology, Writing – review & editing, Project administration. TA: Investigation, Methodology, Project administration, Writing – review & editing.
